# Induction of Innate Inflammatory Pathways in the Corneal Epithelium in the Desiccating Stress Dry Eye Model

**DOI:** 10.1167/iovs.64.4.8

**Published:** 2023-04-10

**Authors:** Zhiyuan Yu, Ghasem Yazdanpanah, Jehan Alam, Cintia S. de Paiva, Stephen Pflugfelder

**Affiliations:** 1 Department of Ophthalmology, Baylor College of Medicine, Houston, Texas, United States

**Keywords:** dry eye, corneal epithelium_,_ NF-kB, inflammasome, IL-1β, IL-18

## Abstract

**Purpose:**

To determine whether 24-hour exposure to the desiccating stress (DS) dry eye model induces NF-kB and NLRP3 inflammasome pathways in the mouse cornea epithelium.

**Methods:**

Six- to 8-week-old C57BL/6J mice were housed under normal humidity (nonstressed) or subjected to DS from a drafty, low-humidity environment combined with subcutaneous scopolamine four times/day for one day to suppress tear production (DS1). Cornea whole mounts were prepared for immunofluorescent staining, or the corneal epithelium was scraped for NF-kB p-p65 ELISA, Western blot, or real-time PCR to detect NF-kB and inflammasome pathway proteins and gene transcripts, respectively.

**Results:**

NF-kB phospho-p65 protein, nuclear NF-kB p-p65, and expression of the NF-kB inducible cytokines (IL-12a, IL-12b, and lymphotoxin b [Ltb]) and chemokine (CCL-2) genes were significantly increased in DS1 compared to nonstressed control. NLRP3 protein and RNA transcripts significantly increased in DS1. NLRP3 and Caspase-1 immunostaining increased in the cornea epithelium at DS1. At DS1 there was no change in IL-18 and a decrease in IL-1β mRNA transcripts; however, levels of bound and total IL-18 protein increased at DS1, and the level of mature IL-1β increased from DS1 to DS5.

**Conclusions:**

These findings indicate innate NF-kB and NLRP3 inflammasome inflammatory pathways are induced in the corneal epithelium within one day in the DS dry eye model. NF-kB activation was associated with increased expression of inflammatory mediators involved in dry eye. Induction of these pathways is accompanied by increased bound/total IL-18 and mature IL-1β.

The cornea is subjected to microbial, desiccating, and osmotic stresses that can stimulate production of inflammatory mediators by the corneal epithelium.^1^^–^^4^ These mediators can support an immune response to pathogens, but in the case of dry eye, they may have deleterious effects on corneal smoothness, barrier function, and the intraepithelial nerve plexus.^1^^,^^5^

Stimulated expression of inflammatory cytokines is often mediated by activation of stress/inflammatory cell signaling pathways. NF-kB and the NLRP3 inflammasome are two key pathways that regulate expression and or activation, respectively, of dry eye–associated inflammatory mediators and have been found to be induced by dryness or high osmolarity.^6^^,^^7^ Treatment with an NF-kB inhibitor was found to prevent disruption of immune tolerance in the desiccating stress (DS) dry eye model, suggesting that NF-kB activation could be an initiating event in the dry eye inflammatory cascade.^6^ NLRP3 expression increased in cultured retinal pigment epithelial cells via the P_2_Y_1_ receptor after exposure to a high concentration of NaCl in the media.^8^ Similarly, hyperosmolar media stimulated expression of inflammasome components NLRP3, apoptosis-associated speck-like protein containing a caspase recruitment domain (ASC), and caspase-1 in cultured cornea epithelium via TRPM2 channel activation.^9^ The purpose of this study was to determine whether short-term exposure to DS induces NF-kB and the NLRP3 inflammasome pathways in the mouse corneal epithelium.

## Methods

### Animals

The animal protocol for this study was designed according to the ARVO Statement for the use of Animals in Ophthalmic and Vision Research and was approved by the Institutional Animal Care and Use Committee at Baylor College of Medicine (Protocol AN-2032). Female C57BL/6J (B6) mice aged six to eight weeks were purchased from Jackson Laboratories (Bar Harbor, ME, USA).

### Dry Eye Models

DS was induced by inhibiting tear secretion by subcutaneous injection of 0.5 mg/0.2 mL scopolamine hydrobromide (Sigma-Aldrich, St. Louis, MO, USA) in alternating hindquarters four times a day (8 AM, 11 AM, 2 PM, and 5 PM) and housing in a cage with a perforated plastic screen on one side to allow airflow from a fan placed 6 inches in front of it for 16 hours/day for one day. Room humidity was maintained at 20% to 30%. Separate groups of mice were treated with DS alone (DS1) or subcutaneous scopolamine (Scop) alone for one day. Control mice were maintained in a nonstressed (NS) environment at 50% to 75% relative humidity without exposure to an air draft.

### Whole-Mount Immunofluorescent Staining and Confocal Microscopy

Corneas were excised and fixed in 100% methanol for 20 minutes at −20°C followed by washing with Hanks’ buffered saline solution (HBSS) for 3 × 5 minutes with gentle shaking at room temperature (RT). Tissues were permeabilized with 0.4% Triton X-100 in HBSS for 30 minutes at RT and gentle shaking. Goat serum 20% (Sigma-Aldrich) diluted in HBSS was used for one hour blocking at RT. Subsequently, the cornea samples were incubated with primary antibodies (Supplementary Table S1) diluted in 5% goat serum in HBSS at the stated concentrations overnight at 4°C with gentle shaking at dark. The samples were then washed with 0.4% Triton X-100 for 3 × 6 minutes at RT with gentle shaking, followed by incubation with secondary antibodies (Supplementary Table S1) diluted in 5% goat serum/HBSS for one hour at RT with gentle shaking and light protection. The samples were then washed for 3 × 10 minutes with 0.4% Triton X-100 in HBSS, and then counterstained with Hoechst (1:500 in HBSS) for nuclei staining (30 minutes at RT and dark with gentle shaking). The samples were washed 3 × 5 minutes with HBSS, mounted on slides, and flattened with coverslips. Immunofluorescent staining in whole-mount cornea samples was visualized using a laser scanning Nikon confocal microscope (Nikon A1 RMP; Nikon, Melville, NY, USA) and 0.5 µm Z-step. The captured images were processed using NIS Elements Advanced Research (AR) software version 4.20 (Nikon).

### Western Blot

Corneal epithelial cells from non-stressed, DS1 and DS5 mice were scraped and placed in cell lysis buffer (cat. no. 895347; R&D Systems, Minneapolis, MN, USA). Protein concentration was measured using a micro-BCA protein assay (cat. no. 23235; Thermo Fisher Scientific, Waltham, MA, USA) as described.^10^ Fifty micrograms of protein for each sample was resuspended in SDS sample buffer, boiled for five minutes, and analyzed on 4% to 15% mini-protean TGX stain-free gels (cat no. 4568084; Bio-Rad, Hercules, CA, USA). The proteins were transferred to a polyvinylidene difluoride membrane (cat. no. 170–4157, Bio-Rad). The blots were incubated with an anti-NF-kBp-p-65 (cat. no. ab106129, phosphor- S276, 1:1000; Abcam, Cambridge, MA, USA), anti-β actin antibody (cat. no. A5441, 1:1000; Sigma-Aldrich), anti-NLRP3 (cat. no. NBP1-77080SS, 1:1000; Novus Biologicals, Littleton, CO, USA), anti-Caspase-1 (cat. no. 22915-1-AP, 1:1000; Proteintech, Rosemont, IL, USA), anti-IL-18 (PA5-79481, 1:1000; Thermo Fisher Scientific), IL-18BP (cat. no. A6445, 1:1000; ABclonal Technology, Woburn, MA, USA), or IL-1β (cat. no. 12426, 1:1000; Cell Signaling Technology, Danvers, MA, USA) overnight. After secondary antibody incubation, the signals from antigen–antibodies complexes were developed with ECL plus Western Blotting Detection kit (ECL, cat. no. RPN2106; GE Healthcare, Chicago, IL, USA). Images were taken using ChemiDoc Touch Imaging System (ChemiDoc Touch Imaging System; Bio-Rad), and band densities were measured by Bio-rad software (Image lab, version 6.0; Bio-Rad).

### RT-PCR

After euthanasia, the corneal epithelium was scraped, and total RNA was extracted using a QIAGEN RNeasy Plus Micro RNA isolation kit (cat. no. 74034; Qiagen, Hilden, Germany) according to manufacturer's instructions. The cDNA was synthesized using the Ready-To-Go^−^You-Prime-First-Strand kit (cat. no. 279264D-100; GE Healthcare, Pittsburgh, PA, USA). Quantitative real-time PCR was performed with specific Taqman probes (Life Technologies, Grand Island, NY, USA) for myd88 (Mm00440338_m1), il1b (Mm00434228_m1), il12a (Mm00434165_m1), il12b (Mm00434174_m1), lymphotoxin b (ltb) (Mm00434774_g1), ccl2 (Mm00441242_m1), nlrp3 (Mm00840904-m1), casp1 (Mm00438023_m1), il18 (Mm00434225_m1), il18bp (Mm00456733_m1), hprt-1 (Mm00446968_m1). The *hprt-1* gene was used as an endogenous reference for each reaction. The results of real-time PCR were analyzed by the comparative CT method.

### NF-kB TransAM Assay

Corneal epithelium was scrapped with a dulled blade and nuclear protein extraction was performed according to the manufacturer's instructions. NF-κB p65 activation was measured by a TransAM NF-κB p65 kit that specifically quantifies phosphorylated NF-κB p65 (Active Motif, Carlsbad, CA, cat. no. 40596). Nuclear extracts from non-stressed and DS1 corneas were added to wells of a 96-well plate with immobilized oligonucleotide containing an NF-κB consensus binding site. The activated p65 in the nuclear extract binds to the oligonucleotide. After incubation with specific anti-p-p65 antibodies, horseradish peroxidase conjugated secondary antibodies provided a sensitive colorimetric readout at 450 nm using a colorimetric plate reader (Tecan Infinite M200, Magellan V6.55 software; Tecan, Männedorf, Switzerland).

### Statistical Analyses

GraphPad Prism 9.0 software (GraphPad, San Diego, CA, USA) was used for statistical analyses. Based on normality, parametric Student *t* or nonparametric Mann–Whitney U tests were performed for between-group statistical comparisons with an alpha of 0.05 using. ANOVA with Tukey post-hoc multiple comparisons were used for analyzing components of the dry eye model.

## Results

These studies evaluated the effects of 24 hours of the desiccating stress dry eye model on induction of the NF-kB and NLPR3 inflammasome pathways in the corneal epithelium.

### NF-kB Activation

**Figure 1.**
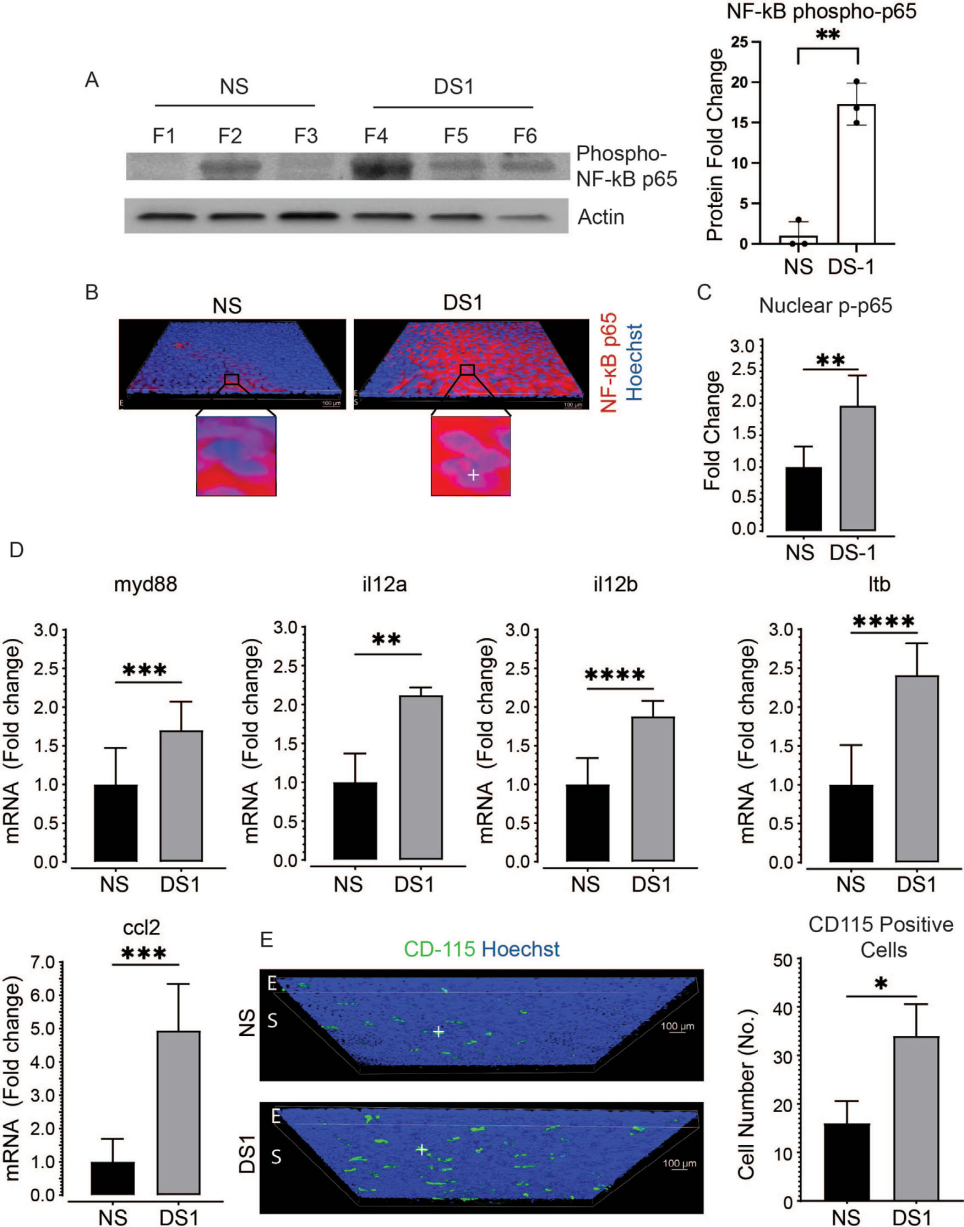
Effects of DS1 on NF-kB and related factors**. (A)** Compared to NS control, increased levels of phospho-NF-kB p65 (p-p65) were detected at DS1 by Western blot. *Bar graph* to the right shows p-p65 band densities normalized to beta-actin (*n* = 3 cornea per group, ** fold change *P* ≤ 0.01). **(B)** NF-kB p65 immunostaining (*red*) in epithelium in representative whole-mount corneas. Sporadic cytoplasmic staining and rare nuclear staining is noted in the NS control and diffuse cytoplasmic and nuclear staining is seen in the DS1 samples (*n* = 3 cornea per group). Nuclei stained blue with Hoechst dye are highlighted with a crosshair in the DS1 group. **(C)** Increased p-p65 in the nuclear fraction of DS1 corneal epithelial cells using a TransAM DNA binding ELISA (*n* = 5 per group, ** fold change *P* ≤ 0.01). **(D)** Expression of NF-kB associated or mediated inflammatory factors that have been found to increase in dry eye, including myd88, cytokines il12a, il12b, ltb, and chemokine ccl2, measured by real time PCR (fold changes **P ≤ 0.01,***P ≤ 0.001, **** *P* ≤ 0.0001). **(E)** Monocyte marker CD-115 (CSR1R) in whole-mount corneal immunostaining from NS and DS. Representative cells positively stained with CD-115 (*green*) are indicated with crosshair in each group. Nuclei are stained blue with Hoechst dye. Cell number comparison in the bar graph to the right (*P = 0.05).

We initially evaluated levels of phosphorylated p65 (p-p65; RelA) an NF-kB component that binds to cognate kB regions in target genes. Compared to NS, increased levels of p-p65 were detected by Western blot (Fig. 1A) and increased NF-kB p65 immunostaining was observed in the cytoplasm and nucleus of the corneal epithelium in whole-mount corneas at DS1 (Fig. 1B). Additionally, we observed increased p-p65 in the nuclear fraction of DS1 corneal epithelial cells using a TransAM DNA binding ELISA (Fig. 1C).

Expression of NF-kB associated or mediated inflammatory factors that have been found to increase or contribute to dry eye pathogenesis, including myd88, cytokines il12a, il12b (a subunit shared by IL-23 and IL-12), ltb and chemokine ccl2, was evaluated by PCR.^11^^–^^15^ Expression of all these genes was significantly increased in the DS1 group (Fig. 1D). CCL2 is a monocyte chemokine and we found an increased number of CD115^+^ monocytes in the DS1 cornea (Fig. 1E).

### NLRP3 Inflammasome

**Figure 2.**
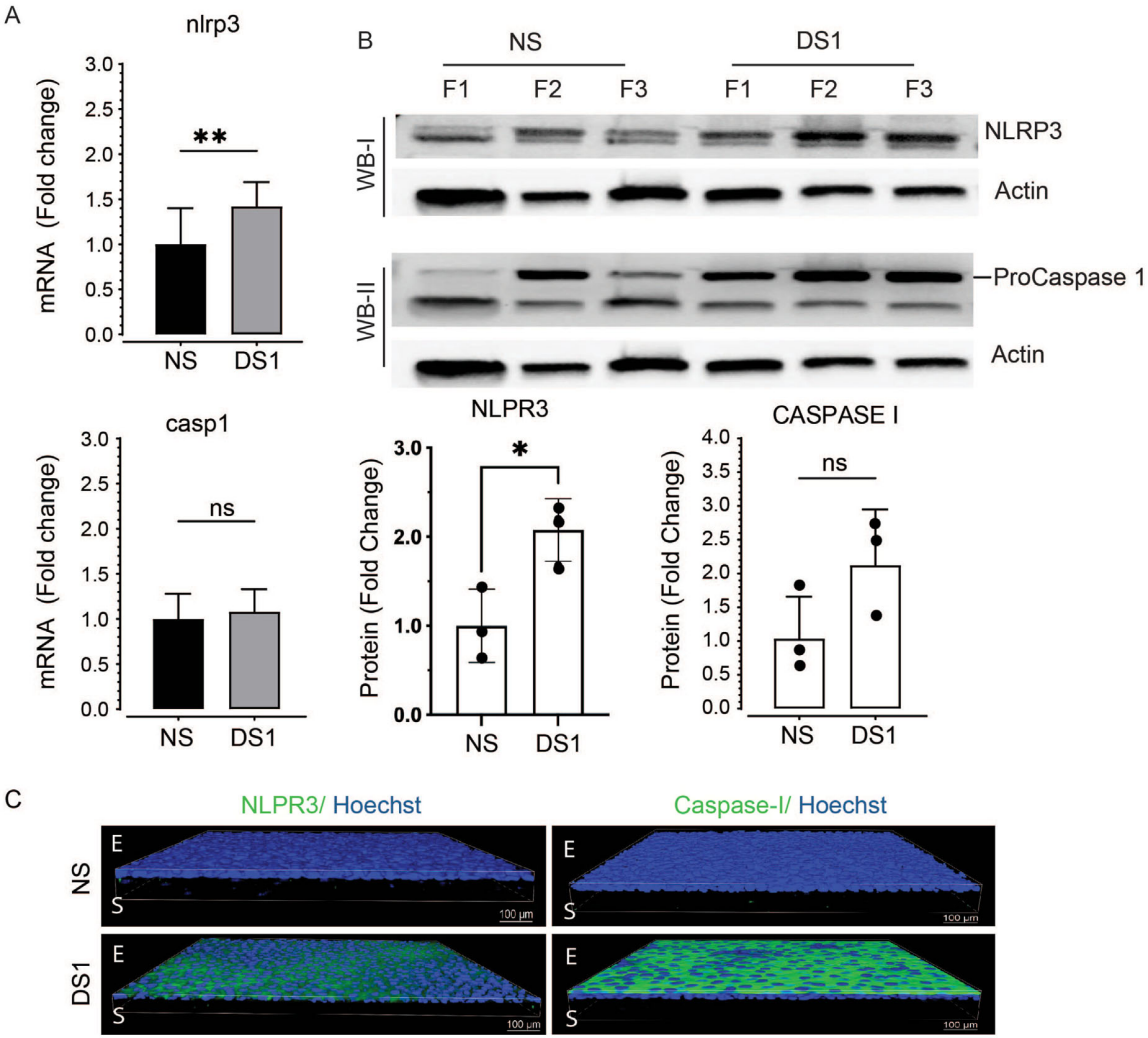
Effects of desiccating stress 1 day (DS1) on NLRP3 inflammasome pathway. **(A)** Expression of inflammasome component genes nlrp3 and casp1 measured by real time PCR at NS and DS1, (fold change **p ≤ 0.01). **(B)** NLRP3 (top) and procaspase-1 (bottom) proteins from NS and DS1 groups detected by Western blot. No 10kDa active caspase-1 band was observed. Bar graphs below shows band densities for NLRP3 (both bands combined) and procaspase-1 normalized to beta-actin (*n* = 3 cornea per group, fold change *P ≤ 0.05). **(C)** Representative NLRP3 and caspase-1 immunostaining in whole-mount corneas from NS and DS, *n* = 3 per group. Nuclei are stained blue with Hoechst dye.

**Figure 3.**
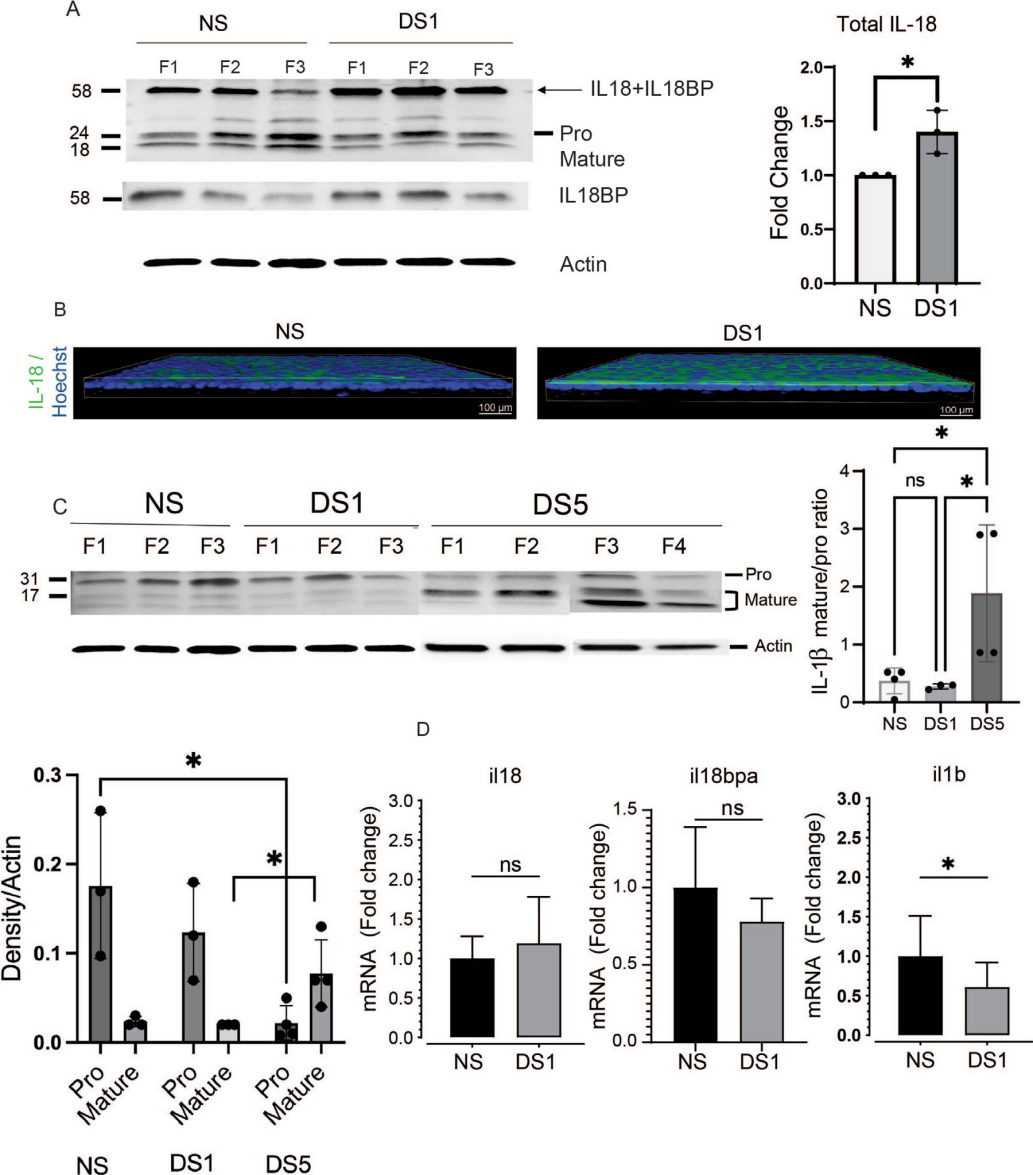
Effects of DS1 on IL-18 and IL-1β. **(A)** Compared to NS control, increased levels of IL-18 binding protein (BP) bound and total IL-18 (pro + mature + bound to IL-18BP) were seen in the DS1 group by Western blot. Identity of the IL-18BP band was confirmed by reacting samples with IL-18BP antibody (*middle blot*). *Bar graph* to the right below shows band densities for all forms of IL-18 (all bands) normalized to beta-actin (*n* = 3 cornea per group, fold change *P = 0.05). **(B)** IL-18 immunostaining in whole-mount corneas from NS and DS1, *n* = 3 per group. Nuclei are stained blue with Hoechst. **(C)** Western blot of pro and mature forms of IL-1β. *Bar graph* below the blot shows pro and mature band densities at each timepoint. Decreased pro-IL-1β compared to NS and increased mature IL-1β compared to DS1 was seen in the DS5 group (*P = 0.05). *Bar graph* to the right below shows pro/mature IL-1β ratios (*n* = 4 corneas per group, fold change *P ≤ 0.05). **(D)** Expression of il18, il18bp and il1β measured by real time PCR at NS and DS1, (fold change *P = 0.05).

Expression of two genes encoding components of the NLRP3 inflammasome (nlrp3 and casp1) was evaluated after exposure to DS for 1 day. The level of nlrp3 transcripts was significantly increased, while there was no change in casp1 (Fig. 2A) at DS1. NLRP3 protein was increased in the DS1 corneal epithelium by both Western blot and whole-mount immunostaining (Figs. 2B, 2C). There was a greater than twofold increase in procaspase-1 protein at DS1 by Western blot (Fig. 2B), but this did not reach statistical significance (*P* = 0.1). Caspase-1 immunostaining was increased in the corneal epithelium (Fig. 2C). Caspase-1 activates pro forms of IL-18 and IL-1β. We found a significant increase in IL-18 bound to IL-18 binding protein (BP), a protein that binds and regulates IL-18 activity,^16^ as well as total IL-18 (pro + mature + bound to IL-18BP) in DS1 by Western blot (Fig. 3A).There was increased IL-18 immunoreactivity in the epithelium of whole-mount DS1 corneas (Fig. 3B). There was no change in levels of pro or mature IL-1β at DS1, but the pro form was decreased at DS5 and the mature form increased on DS5 compared to DS1 (Fig. 3C). The pro/mature IL-1β ratio progressively decreased over five days of DS and was significantly decreased at DS5 compared to NS and DS1 (Fig. 3C). Expression of il18, il18bp genes was unchanged; however, il1b gene expression decreased at DS1 (Fig. 3D).

**Figure 4.**
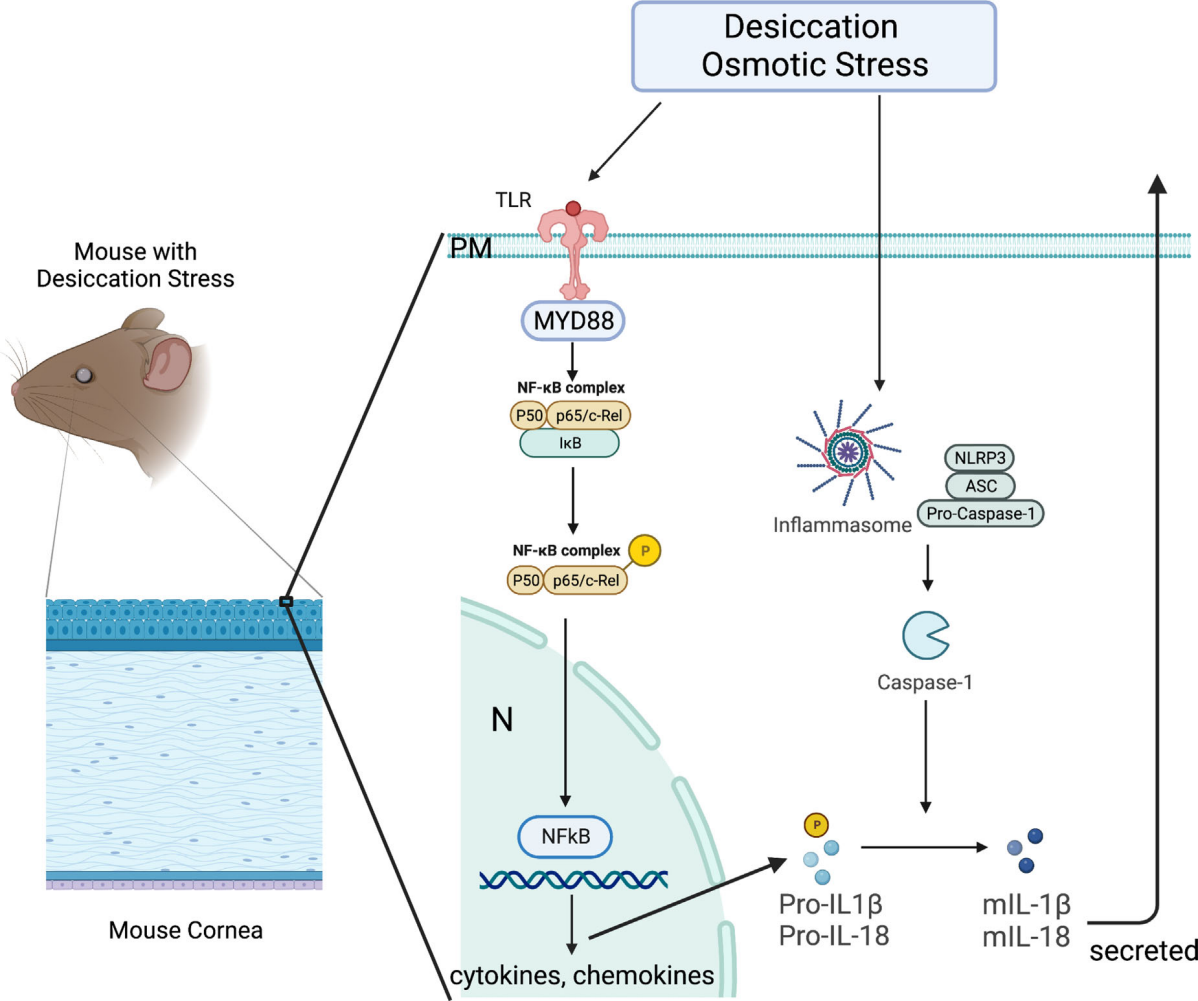
Illustration showing effects of acute desiccating and osmotic stress on NF-kB and NLRP3 inflammasome in the corneal epithelium. On activation NF-kB p65 is phosphorylated and translocates to the nucleus to regulate expression by binding to open kB regions in promoters of inflammatory genes. For the NLRP3 inflammasome, DS1 increased expression of NLRP3 and procaspase-1. Bound and total IL-18 protein increases after 1 day of DS, but increased mature IL-1β was not observed until five days of DS. Mature IL-18 and IL-1β are secreted from the cell (*arrow*).

### Comparison of Dry Eye Model Components in NF-kB and Inflammasome Induction

The standard DS dry eye model combines pharmacological suppression of tear production via systemic administration of the anticholinergic agent scopolamine and exposure to a dry, drafty environment. Systemically administered scopolamine has been found to increase levels of NLRP3 inflammasome components (NLRP3 and caspase-1), as well as IL-1β, IL-18, and caspase-1 activity in various regions of the brain.^17^ Additionally, scopolamine was reported to increase TNF-α and levels of phosphorylated NF-kB in the hippocampus.^18^ To determine whether short-term scopolamine or DS alone can activate NF-kB and inflammasome, we compared the effects of each component of our standard DS model to the full model. Mice received one of three treatments, subcutaneous scopolamine (Scop), exposure to the desiccating environment for 1 day (DS1) or combined scopolamine and desiccating environment (Scop + DS) for one day and levels of NF-kB and inflammasome components were measured. The levels of NF-kB p-p65 protein in the Scop + DS group was higher than the Scop group by Western blot (Supplementary Fig. S1A) and was higher than Scop or DS alone in the nuclear fraction of corneal epithelia by ELISA (Supplementary Fig. S1B). There was no difference in levels of NLRP3 between groups (Supplementary Fig. S1C; however, procaspase 1 was greater in the Scop + DS group than the Scop or DS groups (Supplementary Fig. S1D). These studies show that NF-kB and NLRP3 inflammasome components are only induced in response to dry eye created with the full DS model.

## Discussion

We found activation and nuclear translocation of NF-kB p65 (RelA) and increased expression of NLRP3 in the corneal epithelium after 24 hours of the desiccating stress dry eye model. The corneal epithelial cells in dry eye are subjected to several stresses, including increased tear osmolarity and increased levels of free radicals.^19^^–^^23^ Exposure of cells to osmotic stress has been found to cause clustering and internalization of cell membrane receptors (cytokine and Toll-like receptors) leading to activation of inflammatory signaling pathways.^24^ Increased expression of toll-like receptors (TLR) 2, 3, 5 and 9 in the corneal epithelium has been previously reported in the DS dry eye model.^25^ We have found that exposure of primary cultured corneal epithelial cells to osmotic stress increases caspase-1 activity and expression of NLRP3, IL-1β and IL-18.^21^ The NF-kB pathway regulates production of numerous inflammatory mediators that have been found to be directly or indirectly involved in pathogenesis of dry eye disease,^15^ while activation of the NLRP3 inflammasome activates IL-18 and IL-1β, two key inflammatory cytokines.^26^ IL-1β in particular is known for its wide ranging biological activity on multiple cell types.^27^

Consistent with increased NF-kB activation, we found increased expression of myd88, an adaptor molecule for most TLRs that mediates induction of inflammatory cytokines via NF-kB.^15^ Increased expression of NF-kB regulated cytokines (il12a, il12b, and ltb) and the chemokine ccl2 that are involved in dry eye pathogenesis was also observed.^12^^–^^14^ IL-12a (partnered with IL-12b) is a component of IL-12, a cytokine that stimulates IFN-γ production by conventional T cells and NK cells.^28^^,^^29^ Together with p19, IL-12b is also serves as a heterodimeric component of IL-23, a cytokine that stimulates IL-17 production by CD4 and γδ T cells.^28^ Desiccation induced expression of both IL-12 subunits may be a key initiating step for stimulated production of IFN-γ and IL-17 by lymphocytes, cytokines that cause dysfunction and disease of the ocular surface epithelium in dry eye.^30^^–^^32^ CCL2 is a monocyte chemokine that stimulates recruitment of inflammatory monocytes to the conjunctiva in response to desiccating stress.^13^^,^^33^ Consistent with the increase in CCL2 expression at DS1, we found an increased number of CD115^+^ monocytes in the cornea. These findings support the use of pharmacological agents that suppress NF-kB to blunt desiccation induced inflammation. One mechanism of action of corticosteroids is suppression of NF-kB mediated gene transcription,^34^^,^^35^ and corticosteroids have been found to suppress desiccation induced corneal epithelial disease and gene expression in humans and mice.^1^^,^^36^

The NF-kB and Inflammasome pathways work in tandem to stimulate inflammation (Fig. 4)^26^ and our studies provide evidence of NLRP3 inflammasome activation after one day of DS. The NLRP3 inflammasome is activated by particulates, ion flux, osmotic stress, and free radicals.^7^^,^^21^^,^^26^ Models have predicted that the corneal epithelial cells are exposed to spikes in osmolarity as high as 900 mOsm/L in areas of tear break-up in dry eyes.^37^ Inflammasome induction leads to activation of caspase-1, an enzyme that cleaves and activates the precursor forms of the innate cytokines IL-1β and IL-18.^26^ Increased levels of caspase-1 have been detected in tears of dry eye patients.^38^ We found increased NLRP3 at the gene and protein levels at DS1. Caspase-1 gene expression was unchanged and protein levels showed a trend toward significance at DS1. Levels of IL-18 RNA transcripts did not change at DS1, and unexpectedly, IL-1β transcripts were decreased at DS1. The level of bound and total IL-18 protein (which includes the pro, mature, and bound forms) was increased; however, there was no change in IL-1β pro, mature, or pro/mature ratio at DS1. To determine whether more prolonged exposure to DS would increase pro or mature IL-1β, we evaluated levels of pro and mature forms and the pro/mature ratio at DS5. By that timepoint, mature IL-1β was significantly increased compared to DS-1. This suggests that it takes longer than 24 hours of DS for mature IL-1β to increase in the corneal epithelium. Another factor contributing to these findings is that mature IL-1β is secreted from the cell, and in this mouse model it is not possible to measure all the mature IL-1β because only levels in the cytosol are measured. The findings in this study are consistent with those in our previous studies using the DS dry eye model that show levels of IL-18 and IL-1β transcripts do not significantly change over 10 days of DS.^3^^,^^32^^,^^39^ In contrast to the mouse DS model, we reported that both IL-1β and IL-18 expression significantly increases within four hours of osmotic stress in primary cultured corneal epithelial cells.^21^^,^^40^ A possible explanation for the disparity between the in vivo and in vitro findings is that the stratified corneal epithelium undergoes cell death by apoptosis and pyroptosis, and the apical epithelial cells that are exposed to the maximal stress are shed.^41^^–^^43^ It may take time for IL-1β protein present in the cytoplasm of subapical epithelial cells to undergo cleavage by activated caspase-1. This is consistent with our previously reported finding that IL-1β protein concentration in tears is significantly increased after five and 10 days of DS, and the shed cells may be a source of this IL-1β.^3^ IL-1β and IL-18 have been found to contribute to dry eye–induced inflammation. IL-18 (originally termed IFN-γ inducing factor^44^) is required for full expression of IFN-γ by NK and conventional T cells.^45^^,^^46^ IL-1β has multiple activities in dry eye including stimulating expression of other innate cytokines and chemokines, matrix metalloproteinase-9 (MMP-9) a protease that disrupts corneal barrier function, and IL-17 production by γδT cells.^14^^,^^39^^,^^47^

A weakness of this study is use of the anticholinergic agent scopolamine to suppress tear production in the established DS dry eye model because systemically administered scopolamine has been found to increase levels of phosphorylated NF-kB, as well as NLRP3 inflammasome components (NLRP3 and caspase-1) in various regions of the brain.^17^^,^^18^ We performed an experiment to evaluate whether scopolamine or DS alone activate components of the NF-kB and inflammasome pathways. Increased levels of p-p65NF-kB protein and nuclear protein and caspase-1 were found in the group subjected to the full DS model but not in the scopolamine or DS alone groups. These findings indicate that short-term system administration of scopolamine does not induce components of these pathways in the corneal epithelium and the effects of scopolamine are secondary to pharmacological inhibition of tear production. When we developed the DS model used in this study, we compared the relative effectiveness of scopolamine-induced tear suppression alone, desiccating environmental stress (low humidity, drafty environment) alone or the combination of scopolamine and desiccating stress on their ability to cause clinically apparent corneal epithelial disease.^48^ Minimal corneal epithelial dye staining was observed with scopolamine alone, whereas combined scopolamine and environmental stress produced corneal staining consistent with clinically significant dry eye. Innate inflammatory mediators, such as MMP-9, are important in the pathogenesis of corneal barrier disruption in dry eye.^49^ MMP-9 and some cytokines that induce it (e.g., IL-1β) are modulated by NF-kB and inflammasome pathways.^15^ Evidence from these experiments indicates that the combination of reduced tear production and a desiccating environment is capable of inducing production of these inflammatory mediators in the corneal epithelium hours. These findings are relevant to researchers in the field because the DS dry eye model is validated and widely used.^50^^,^^51^ Use of anticholinergic medications has been identified as a significant risk for developing dry eye, and use of these might make dry eye patients more susceptible adverse environments that are associated with dry eye flares.^52^ Increased production of cytokines IL-1 and IL-18 can amplify the immune reaction of dry eye by stimulating production of cytokines IFN-γ and IL-17 by lymphocytes that cause ocular epithelial disease, including goblet cell loss and corneal barrier disruption.^14^^,^^32^^,^^53^

## Supplementary Material

Supplement 1

Supplement 2
