# Supplementary material for: Induction of Innate Inflammatory Pathways in the Corneal Epithelium in the Desiccating Stress Dry Eye Model
Source: Invest Ophthalmol Vis Sci. 2023 Apr 10;64(4):8. doi: 10.1167/iovs.64.4.8 (PMC10103726; doi:10.1167/iovs.64.4.8)
Supplement: Supplement 1 [file iovs-64-4-8_s001.pdf]

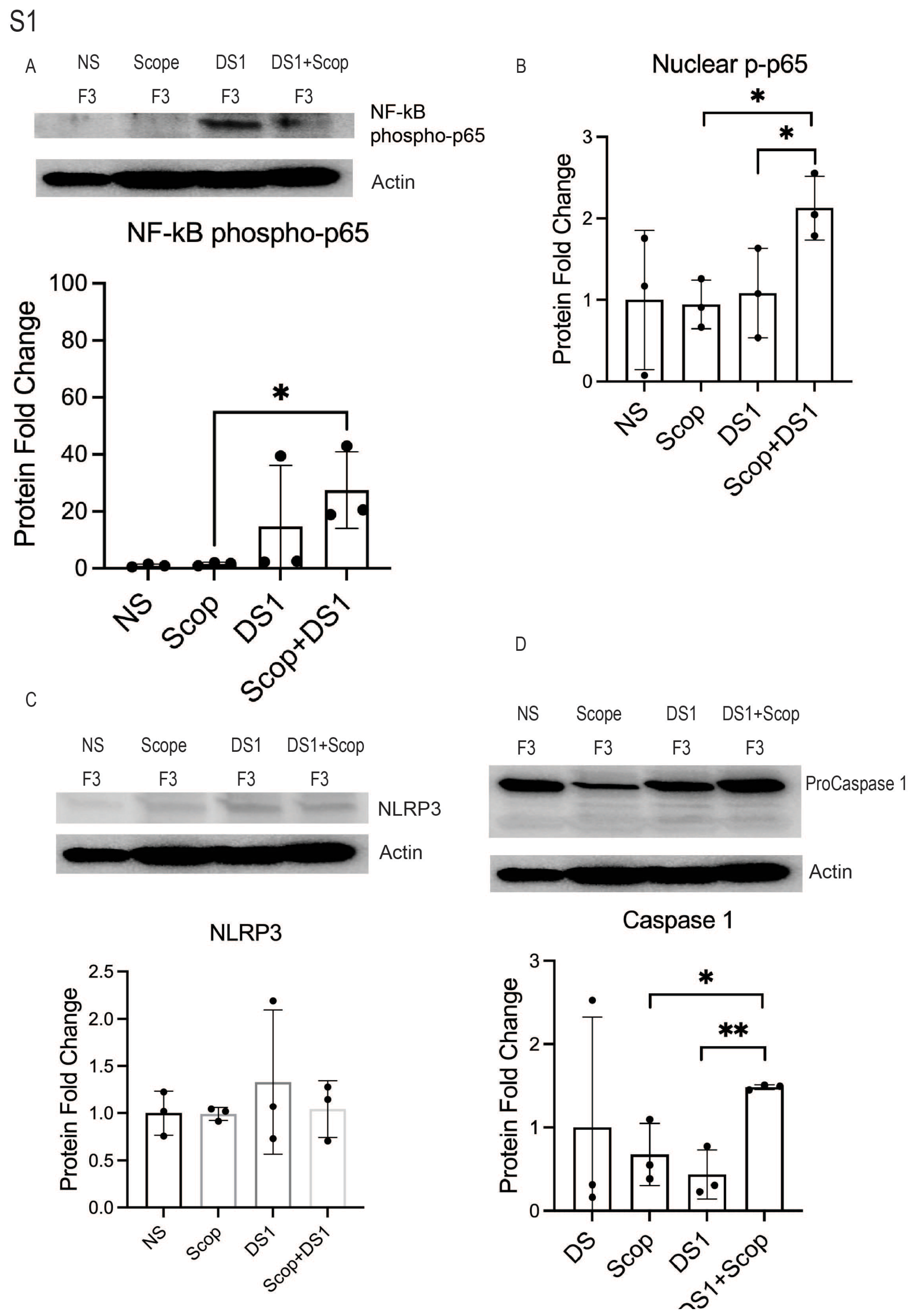

Supplemental Fig 1. Effects of dry eye model components [scopolamine (scop); desiccating stress (DS1), DS1+scop] compared to the non-stressed (NS) group on NF-kB and Inflammasome activation. A. NF-kB p-p65 western blot of samples from 4 experimental groups. Bar graph shows p-p65 band densities normalized to beta-actin (n= 3 samples per experimental group). Levels of phospho-NF-kB p65 (p-p65) were significantly higher in DS1+Scop compared to Scop alone (fold change *p<0.05); B. NF-kB p-p65 DNA binding ELISA of samples from 4 experimental groups. Levels of nuclear protein were significantly higher in Scop+DS1 compared to the Scop or DS1 groups (n=3/group, fold change *p<0.05); C. NLRP3 western blot of samples from 4 experimental groups. Bar graph shows NLRP3 band densities normalized to beta-actin (n= 3 cornea per group); D. Caspase 1 western blot of samples from 4 experimental groups.
Bar graph to the right shows procaspase 1 band densities normalized to beta-actin (n= 3 cornea per group, procaspase 1 was significantly higher in DS1+Scop compared to the Scop and DS1 groups alone (fold change *p<0.05, ** p<0.01)
