# Supplementary material for: Induction of Innate Inflammatory Pathways in the Corneal Epithelium in the Desiccating Stress Dry Eye Model
Source: Invest Ophthalmol Vis Sci. 2023 Apr 10;64(4):8. doi: 10.1167/iovs.64.4.8 (PMC10103726; doi:10.1167/iovs.64.4.8)
Supplement: Supplement 2 [file iovs-64-4-8_s002.pdf]

Supplementary Table 1, primary and secondary antibodies

Supplementary Table 1							
**Antibody Type**	**Target**	**Clone**	**Host**	**Reaction**	**Conjugation**	**Company (Catalog No.)**	**Dilution**
Primary	Csf1r (CD115)	YEYE311	Rat	Mouse	No Conjugation	Abcam, (ab271294)	1:50
Primary	NF-κB P65	Polyclonal	Rabbit	Mouse	CoraLite®594 Fluorescent Dye	Proteintech (CL594-10745)	1:30
Primary	NLPR3	Polyclonal	Rabbit	Mouse	No Conjugation	Novus (NBP1-77080SS)	1:30
Primary	Caspase-I	B0-6	Mouse	Mouse	No Conjugation	ThermoFisher (MA5-23909)	1:30
Primary	IL-18	Polyclonal	Rabbit	Mouse	No Conjugation	ThermoFisher (PA5-79481)	1:30
Secondary	IgG (H+L)	Polyclonal	Goat	Rabbit	Alexa Fluor 488	Jackson ImmunoResearch (111-545-003)	1:1000
Secondary	IgG (H+L)	Polyclonal	Goat	Rat	Alexa Fluor 488	Jackson ImmunoResearch (112-545-003)	1:1000
Secondary	IgG (H+L)	Polyclonal	Goat	Mouse	Alexa Fluor 488	Jackson ImmunoResearch (115-545-146)	1:1000
